# The Effects of Congruence Between Person and Environment on Innovation Performance in Ports

**DOI:** 10.3389/fpsyg.2021.732660

**Published:** 2021-11-24

**Authors:** Daokui Jiang, Teng Liu, Zhuo Chen, Xiaoyan Zhang, Su Wang, Tianci Huang, Lei Ning

**Affiliations:** ^1^Business School, Shandong Normal University, Jinan, China; ^2^School of Innovation and Entrepreneurship, Shandong University, Qingdao, China; ^3^School of Entrepreneurship Education, The Open University of China, Qingdao, China; ^4^School of Economics, Ocean University of China, Qingdao, China; ^5^School of Economics and Management, Qingdao University of Science and Technology, Qingdao, China

**Keywords:** person-environment fit, need-supply (N-S) fit, demands-abilities, polynomial regression analyses, response surface analysis

## Abstract

The projected growth and rapid technological development in maritime transportation will create demand for a newly skilled and motivated workforce in the port sector. Thus, it is important for ports to attract, recruit and retain talented employees to promote innovation and enhance competitive advantages. This manuscript focuses on the welfare and talent of port staff from the perspective of person-environment (P-E) fit. Using polynomial regression with response surface analysis, this study explores the effect of P-E fit on job satisfaction, work engagement and innovation performance, and bootstrapping is applied to confirm the mediating roles of job satisfaction and work engagement in the relationship between P-E fit and innovation performance. Results show that (1) need-supply (N-S) fit and demands-abilities (D-A) fit improved port employees’ job satisfaction, work engagement and innovation performance, and the impacts on work engagement and innovation performance show an inverted “U” and “U” shape, respectively; (2) D-A fit is more important when job satisfaction plays a mediating role; and (3) N-S fit makes a greater contribution when work engagement mediates the effect of P-E fit on the innovation performance. These findings contribute to P-E fit research as well as to human resource management practices in ports.

## Introduction

The COVID-19 pandemic had a significant impact on global supply chains at every level, including the port and shipping industry. Port and terminal operations were heavily affected by the overall reduction in cargo volume due to the pandemic (PwC, 2021). Fortunately, world trade is showing signs of recovery, with most economic sectors resuming activities (International Monetary Fund [IMF], 2020). In addition, maritime transportation will continue to dominate freight; therefore, the demand for associated maritime services will increase in the long run (International Transport Forum [ITF], 2019). Shipping through steamships and containerization has always been a key initial factor driving world trade development, while digitalization and innovation will be another important factor in the future.

Innovation in port sectors is developing rapidly, but the tension between attempts to innovate and the ability to achieve the expected goals needs to be resolved (Chen et al., 2019; Koukaki and Tei, 2020). Engendering innovative work behavior among employees is one of the best ways to promote innovation (Andrew, 1986), and employees’ innovation performance plays a crucial role in achieving organizational objectives and high firm performance (Klomp and Leeuwen, 2001; Darroch, 2005; Karatepe and Sokmen, 2006). Thus, organizations’ ability to attract, recruit and retain talented employees is crucial to corporate success, and being in the right job and the right organization is an important contributor to a high quality of work life for employees (Alniacik et al., 2013). The projected growth of maritime transportation and rapid technological developments will significantly change employment patterns in the maritime industry and create the need for a newly skilled, competent and motivated workforce (Cicek et al., 2019). Therefore, port and terminal companies need to focus on the welfare and talent of their employees.

Person-environment fit (P-E fit) is central to research in human resource management (Holland, 1997; Edwards et al., 1998) and directly impacts job satisfaction, work motivation, organizational citizenship behavior and performance, organizational commitment, and professional mental health (Kristof-Brown et al., 2005; Edwards et al., 2006; Shen et al., 2018). Many scholars have investigated the relationship between P-E fit and innovation performance from the perspective of different forms of P-E fit, such as person-job (P-J) fit and person-organization (P-O) fit (Tak, 2011). Few scholars operationalize P-E fit in line with need-supply (N-S) fit and demands-abilities (D-A) fit, and they have not yet theoretically modeled the relationship among P-E fit, job satisfaction, work engagement, and the innovation performance of employees (Lauring and Selmer, 2018). Thus, this study’s first aim is to identify the mechanism influencing the role of P-E fit in employees’ innovation performance in port groups from the perspectives of N-S fit and D-A fit. In addition, unlike previous studies reporting linear positive impacts of P-E fit on innovation performance, this study explores the relationship among P-E fit, job satisfaction, work engagement and employees’ innovation performance in ports by polynomial regression with response surface analysis. The results of this study could provide references for port management practices to improve employees’ innovation performance and organizational innovation performance.

This manuscript is organized as follows: a literature review and hypotheses are presented in section two; section three outlines the research methods, including the data sources, variable measurement and hypothesis development methods; section four comprises the results and analysis; and section five presents the conclusions and implications.

## Theoretical Background and Hypotheses

The concept of P-E fit has long been prevalent in the vocational behavior literature as well as the management literature (e.g., Edwards, 1994; Kristof, 1996). P-E fit is defined as the match between a person and his or her environment (French et al., 1982; Muchinsky and Monahan, 1987; Chatman, 1989; Kristof, 1996; Edwards et al., 2006; Edwards, 2008), and N-S fit, D-A fit and supplementary fit are three dominant topics in P-E fit research (Kristof, 1996; Edwards et al., 2006; Goetz et al., 2021). N-S fit refers to the match between environmental supply and the psychological needs (i.e., desires, values, goals) of the employee, and D-A fit is the compatibility between the demands of the environment and individuals’ personality, knowledge, skills, and abilities (Kristof-Brown, 2000; Edwards et al., 2006). N-S fit and D-A fit are two types of complementary fit that capture the degree of one to fulfill the requirements of the other. Supplementary fit refers to the similarity between the person and the environment (Edwards et al., 2006). The increasing digitalization and automation in the shipping industry will make different and more technically advanced knowledge and expertise a necessity (Cicek et al., 2019), and supplementary fit occurs if an organization employs a person with skills that are similar to those already widely possessed in its workforce; it is most typically investigated by examining the value congruence between employees and organizations (Cable and Edwards, 2004). Therefore, this study focuses on the effects of complementary fit on employees’ innovation performance. Since employees who have the requisite skills, abilities and knowledge to be competent at their jobs likely do not share the organization’s values and *vice versa* (Lauver and Kristof-Brown, 2001), N-S fit and A-D fit are examined separately.

### Person-Environment Fit and Job Satisfaction

In applying P-E fit concepts, levels within the environment can be considered to provide a better foundation for analysis (Edwards, 2008). P-E fit research is dominated by an emphasis on individual and personal satisfaction (Gul et al., 2018). Job satisfaction is defined as a positive emotional state due to the employee’s appraisal of the job or job experience (Locke, 1976). Many scholars point out that N-S fit and D-A fit matter in terms of job satisfaction (Rounds et al., 1987; Rice et al., 1989; Kristof-Brown et al., 2005; Edwards and Shipp, 2007), and empirical analysis has also revealed that mutual fit between the characteristics of the employee and the requirements of the organization lead to job satisfaction and job commitment (Mulky, 2012; Oh et al., 2014; Andela and Doef, 2018; Rauvola et al., 2020). However, increasing the environmental supply to improve P-E fit does not necessarily promote job satisfaction if individual needs are thoroughly satisfied. Therefore, some scholars think that non-linear models can better describe this relationship (Pee and Min, 2017). In addition, a high level of job satisfaction requires much personal engagement. People may feel frustrated and nervous or even leave the job if they sense an imbalance between investment and reward (Atitsogbui and Amponsah-Tawiah, 2019), which indicates an inverted U-shaped relationship between person-environment fit and job satisfaction. Based on the above findings, the following is proposed:

Hypothesis H1a: N-S fit has a decreasing incremental effect on job satisfaction, that is, a positive linear and negative conic effect.
Hypothesis H1b: D-A fit has a decreasing incremental effect on job satisfaction, that is, a positive linear and negative conic effect.

### Person-Environment Fit and Work Engagement

Work engagement, a positive, fulfilling, work-related emotional state characterized by vigor, dedication and absorption (Schaufeli et al., 2002; Schaufeli and Bakker, 2010), is a step above satisfaction (Robertson-Smith and Markwick, 2020). A sustainable workload, feelings of choice and control, appropriate recognition and reward, a supportive work community, fairness and justice, and meaningful and valued work lead to work engagement (Maslach et al., 2001), and a wide range of human resource practices, including competitive compensation, incentives and rewards, promotion, job security, flexible job design, employee involvement, and information sharing, have significant effects on work engagement (Zhang et al., 2013; Karatepe et al., 2018). P-E fit promotes work engagement (Bakker and Demerouti, 2014; Cai et al., 2018), and empirical evidence has confirmed this relationship (e.g., Lascbinger et al., 2006; Maslach and Leiter, 2008; Shuck et al., 2011). N-S fit still matters when work engagement is viewed as a state of well-being (Schaufeli and Bakker, 2004; Shaw and Gupta, 2004). However, work engagement can be hindered by excessive workload, despite P-E fit playing a motivational role in reducing the negative effects and promoting work engagement (Bakker and Demerouti, 2014). Based on the above analysis, the following are proposed:

Hypothesis H2a: N-S fit has a decreasing incremental effect on work engagement, that is, a positive linear and negative quadratic effect.
Hypothesis H2b: D-A fit has a decreasing incremental effect on work engagement, that is, a positive linear and negative quadratic effect.

### Person-Environment Fit and Employees’ Innovation Performance

Fit may be a viable construct at many levels of analysis, and there are many types of P-E fit, such as person-organization (P-O) fit, person-vocation (P-V) fit, person-group (P-G) fit, and person-job (P-J) fit (Kristof, 1996). P-J fit, which is defined as the fit between an individual’s abilities and the demands of a job (D-A) or between an employee’s desires and the supply of a job (N-S), significantly influences the innovative behavior of employees (Collins and Amabile, 1999). When job characteristics, organizational demands, and resource availability match individuals’ abilities and intrinsic needs, employees are likely to reciprocate and respond more creatively to their situations since they have a high level of commitment to and satisfaction with their jobs (Kristof-Brown et al., 2005; Hon and Rensvold, 2006). P-O fit is defined as the match between the preferences or needs of individuals and organizational systems and structures, reflecting the N-S conceptualization (Kristof, 1996). High P-O fit also helps employees engage in innovative behavior (Verquer et al., 2003; Kristof-Brown et al., 2005; Lee and Wu, 2011; Afsar et al., 2015). Moreover, from the perspective of D-A fit, studies have shown that teams are more effective when members have heterogeneous knowledge, skills, and abilities (e.g., Haythorn, 1968; Shaw, 1981).

Hypothesis H3a: N-S fit has an increasing incremental effect on innovation performance, that is, a positive linear and positive quadratic effect.
Hypothesis H3b: D-A fit has an increasing incremental effect on innovation performance, that is, a positive linear and positive conic effect.

### The Mediating Role of Job Satisfaction in the Relationship Between Person-Environment Fit and Work Engagement

Person-environment fit is related to organizational citizenship behaviors, self-reported teamwork, and work performance (Farzaneh et al., 2014; Oh et al., 2014; Kristof-Brown et al., 2016), and misfit leads to job dissatisfaction (Edwards and Shipp, 2007). People with a high level of job satisfaction are highly engaged in their work (Bakker and Demerouti, 2008; Giallonardo et al., 2010; Alarcon and Lyons, 2011; Bakker, 2011; Cote et al., 2021). For example, P-J fit significantly affects work engagement by influencing employees’ motivational states, which results in experiences of positive feelings (Warr and Ilke, 2012). A good P-O fit makes employees satisfied with their tasks and intrinsically motivated to engage in innovative work behavior more often (Silverthorne, 2004). Based on the above analysis, the following hypothesis is proposed:

Hypothesis H4: Job satisfaction mediates the relationship between P-E fit and work engagement.

### The Mediating Role of Work Engagement in the Relationship Between Person-Environment Fit and Innovation Performance

Work engagement mediates the link between some work-life factors (workload, control, rewards and recognition, community and social support, and perceived fairness and values) and work outcomes (Kular et al., 2008). There is strong evidence that work engagement is a mediator of positive work outcomes (Scrima et al., 2014; Karatepe et al., 2018) and that employees’ performance can be improved by work engagement (Karatepe, 2011; Yalabik et al., 2013; Turner, 2020). For example, Karatepe (2013) found work engagement to be a full mediator of the effects of certain HR practices on job performance and extra-role customer service. Engaged employees often experience positive emotions (Schaufeli and Rhenen, 2006). According to the broaden-and-build theory (Fredrickson, 2001), the positive emotions of engaged employees, such as happiness, joy, and enthusiasm (Bakker and Demerouti, 2008), share the capacity to broaden people’s momentary thought – action repertoires and build personal resources by widening the array of thoughts and actions that come to mind. Joy leads to being creative, and interest fosters the desire to explore new worlds and assimilate new information and experiences. Happy people are more sensitive to opportunities at work, and they are more willing to help others (Cropanzano and Wright, 2001). Employees with better P-O fit are satisfied with their tasks and are intrinsically motivated, and those who are intrinsically motivated are more likely to display innovative work behavior (Jong and Hartog, 2007). Based on the above analysis, the following is proposed:

Hypothesis H5: Work engagement mediates the relationship between person-environment fit and innovation performance.

## Research Methods

### Participants and Procedures

The sample of this study was drawn from employees and their supervisors in port companies using survey methodology, and respondents were guaranteed anonymity and confidentiality. Operational, technical, and administrative posts were included, and the employees were required to have been working full-time for at least 1 year at the time of the study to be eligible for participation. Three representative ports in China, namely, Shanghai Port, Ningbo-Zhoushan Port and Qingdao Port, were selected because they have excellent innovation performance. The specific reasons for selecting these ports are as follows: (1) Shanghai Port has been the largest port in the world in terms of container throughput volume for 5 years; (2) Ningbo-Zhoushan Port is the largest port in China in terms of cargo throughput, and Qingdao Port is the largest port in North China in terms of container throughput volume (Ministry of Transport of the People’s Republic of China [MOT], 2021); (3) Shanghai Port has the largest single fully automated terminal and the most comprehensive automated terminal in the world; and (4) Ningbo-Zhoushan Port is the earliest adopter of remote control automatic gantry cranes without cabs in China, and Qingdao Port has the first fully automated container terminal in Asia (Cariou, 2020).

The following measures were taken in this study to reduce bias: (1) the questionnaire was checked before being sent out; (2) the questionnaire was translated into Chinese, which was the language spoken by the respondents, and three professionals were invited to back-translate it into English to ensure the accuracy of the information; (3) all variables were disordered in order to reduce common method bias (Podsakoff et al., 2003); (4) a sample elimination rule was set, and reverse multiple choice questions were added; (5) samples with short answer times and incomplete data were eliminated; and (6) each completed questionnaire was rewarded with a compensation of eight yuan. A total of 600 questionnaires were sent out, and 402 were ultimately collected, representing a response rate of 67.0%. A total of 379 valid questionnaires were obtained after eliminating those that were invalid, with an efficiency of 94.3%. This study conducts its analysis on the basis of these samples.

### Measurement

Direct measures of fit are used in this manuscript since some researchers have argued that indirect measures may cause the computed similarity (or difference) between individuals and the environment to be responsible for observed relations with an outcome measure (e.g., Edwards and Parry, 1993; Kristof-Brown et al., 2005). Regarding P-E fit, items improved by Cable and DeRue (2002) based on research by Cable and Judge (1996) were used in this manuscript to measure D-A fit, including “The match is very good between the demands of my job and my personal skills,” “My abilities and training are a good fit with the requirements of my job,” and “My personal abilities and education provide a good match with the demands that my job places on me.” Items created by Cable and DeRue (2002) were used to measure N-S fit, including “There is a good fit between what my job offers me and what I am looking for in a job,” “The attributes that I look for in a job are fulfilled very well by my present job,” and “The job that I currently hold gives me just about everything that I want from a job.” Respondents were asked to rate their level of agreement with each statement on a 5-point Likert scale ranging from strongly disagree to strongly agree. The Cronbach’s α of the scale was 0.740 for D-A fit and 0.799 for N-S fit in the current study.

The job satisfaction scale was adopted from Cammann et al. (1983) and includes three items, such as “All in all, I am satisfied with my job.” All items were scored on a 5-point rating scale ranging from 1 (strongly disagree) to 5 (strongly agree). This scale has demonstrated adequate reliability (e.g., McFarlin and Sweeney, 1992). The Cronbach’s α of the scale was 0.860.

Engagement was assessed with the Utrecht Work Engagement Scale (UWES) (Schaufeli et al., 2002). The shortened version, the UWES-9 (Schaufeli et al., 2006), was used to measure work engagement in this manuscript. The nine items in the scale were grouped into three subscales that reflect the underlying engagement dimension: vigor (three items), dedication (three items), and absorption (three items). All items were scored on a 7-point rating scale ranging from 0 (never) to 6 (always). The Cronbach’s α of the scale was 0.896.

Innovation performance was assessed using Janssen’s (2000, 2001) and Janssen and Van Yperen’s (2004) nine-item scale of individual innovation in the workplace, which draws on Kanter’s (1988) work on the stages of innovation. Three items refer to idea generation, three items to idea promotion, and the remaining three to idea realization. High reliability was achieved for the innovation performance scale (The Cronbach’s α of the scale was 0.886).

The reliability of the scales was examined using Cronbach’s alpha, which is the degree of internal consistency and reliability (Gul et al., 2018). The coefficient alpha value provides good estimates, and items are retained when they exceed the minimum standard of 0.70 (Nunnally and Bernstein, 1994). The Cronbach’s α of all the scale were greater than 0.7 in this study, revealing that the scales were reliable for analysis.

### Covariates

To control for the possibility that sociodemographic differences in the predictor and outcome variables might lead to spurious relationships, gender, age (in years), and education were entered as covariates in the analysis following Janssen (2000). Many studies have related these variables to employee work engagement, satisfaction and other positive job attitudes (e.g., Luthans and Thomas, 1989; Martin and Shehan, 1989; Vila et al., 2007; Kular et al., 2008; Boumans et al., 2011; Gonzalez et al., 2016).

### Polynomial Regression With Response Surface Analysis

Correlation or regression analyses are most commonly used to examine the relations between P-E fit and its potential outcomes through direct or indirect measures (Verquer et al., 2003; Kristof-Brown et al., 2005). Polynomial regression with response surface analysis was carried out to test the hypotheses in this study. This method is suitable for testing the correlation between two predictor variables and their mutual consistency with and difference from the outcome variable (Edwards, 1994; Edwards et al., 2006) and has become increasingly popular in multisource feedback research, such as that on self-observer rating discrepancies (Shannock et al., 2010). Polynomial regression with response surface analysis has been applied to human resource management by many scholars in the context of job satisfaction (Harris et al., 2014; Audenaert et al., 2018), work engagement (Yang et al., 2017; Qiu et al., 2020), and innovation performance (Lee et al., 2016).

The polynomial (quadratic) regression equation is given as:


(1) Z=b+0b1X+b2Y+b3X2+b4XY+b5Y2+ε

Response surface methodology is used to interpret and test the features of the graph associated with Equation (1) since the coefficients of a polynomial regression are difficult to interpret directly (Edwards and Parry, 1993). There are three key indicators of response surface technology: the fixed point, main axis, slope, and curvature. Among them, the main axis describes the direction of the response surface on the *X-Y* axis. The first main axis and the second main axis are perpendicular to each other and intersect at a fixed point. The shape of the response surface can be judged according to the spindle. For a convex surface, the curvature along the first major axis is the largest, while the curvature along the second major axis is the smallest; for a concave surface, the curvature along the first major axis is the smallest, and the curvature along the second spindle is the largest. For slope and curvature, the consistency line means that the two measurement indexes are equal and the direction is the same on the XY plane (*X* = Y); the inconsistency line means that the two measurement indexes are the same but the direction is opposite on the XY plane (*X* = −Y). Substituting *X* = Y and *X* = −Y into Equation (1), the formula for calculating the consistency and inconsistency lines is generated:


(2) Z=b+0(b1+b2)X+(b3+b4+b5)X2+ε

(3) Z=b+0(b1-b2)X+(b3-b4+b5)X2+ε

Along the *Y* = X line, the slope is (b_1_+b_2_), and the curvature is (b_3_+b_4_+b_5_); along the *Y* = −X line, the slope is (b_1_−b_2_), and the curvature is (b_3_−b_4_+b_5_). When (b_3_+b_4_+b_5_) and (b_3_−b_4_+b_5_) are negative and have significance, it represents a concave surface (U-shaped) along this line; in contrast, a positive value indicates a convex surface (inverted U-shaped).

In the present study, the outcome variable is employee innovation performance (I), and the two component measures are D-A fit (D) and N-S fit (N). The polynomial (quadratic) regression equation is given as:


(4) $$I=b_{0}+b_{1}\,N+b_{2}\,D+b_{3}\,N^{2}+b_{4}\,N\,D+b_{5}\,D^{2}+\varepsilon$$

## Results and Analysis

### Bivariate Correlation

The statistical information of the respondents is shown in Table 1. These data correspond with the characteristics of the port sector from the perspective of gender, age, and education level.

**TABLE 1:** Statistical information of the respondents.

Covariates	Items	Number	Proportion (%)
Gender	Male	161	42.5
	Female	218	57.5
Age	≤25	254	67
	26–35	99	26.1
	36–50	21	5.5
	≥51	5	1.3
Education	Junior college education and below	150	39.6
	Undergraduate degree	201	53
	Postgraduate degree	28	7.4

Table 2 displays the means, standard deviations, and correlations among the study variables. The results of the bivariate correlation analyses shown in Table 1 indicate that N-S fit, D-A fit, job satisfaction and work engagement are all significantly and positively related to the innovation performance of port employees (*r* = 0.670, *r* = 0.636, *r* = 0.595, *r* = 0.674, and *p* < 0.01).

**TABLE 2:** Means, standard deviations, and correlations among the study variables.

	Variables	Mean	SD	1	2	3	4	5	6	7
(1)	Gender	1.575	0.495	1						
(2)	Age	1.412	0.658	–0.079	1					
(3)	Education	1.678	0.606	0.019	0.081	1				
(4)	N-S fit	4.433	1.249	–0.050	0.092	0.013	1			
(5)	D-A fit	4.681	1.173	–0.061	0.199**	–0.011	0.575**	1		
(6)	Job satisfaction	4.512	1.093	–0.052	0.006	–0.016	0.702**	0.482**	1	
(7)	Work engagement	4.795	1.130	–0.055	0.158**	–0.085	0.684**	0.700**	0.574**	1
(8)	Innovation performance	4.705	1.017	−0.112*	0.097	0.004	0.670**	0.636**	0.595**	0.674*

* and

** indicate p < 0.05 and p < 0.01, respectively.

### Polynomial Regression

The polynomial regression results are shown in Table 3.

**TABLE 3:** Polynomial regression results.

Variables		Job satisfaction		Work engagement			Innovation performance			
				
		Model 1	Model 2	Model 3	Model 4	Model 5	Model 6	Model 7	Model 8	Model 9
Constant term		4.808***	4.821***	4.990***	4.990***	4.420***	4.965***	4.879***	4.013***	3.012***
Gender		–0.040	–0.037	–0.004	–0.004	0.000	–0.140	−0.145*	−0.138*	−0.138*
Age		−0.131**	–0.114	0.063	0.064	0.078	–0.040	–0.034	–0.014	–0.031
Education		–0.029	–0.037	−0.166*	−0.167*	−0.163*	0.010	0.004	0.010	0.047
N-S, *β_1_*		0.553***	0.551***	0.384***	0.356***	0.290***	0.368***	0.410***	0.311***	0.245***
D-A, *β_2_*		0.124***	0.127**	0.432***	0.448***	0.432***	0.327***	0.284***	0.261***	0.163***
N-S^2^, *β_3_*			0.011		–0.017	–0.018		0.061**	0.059**	0.063**
N-S × D-A, *β_4_*			0.034		0.081**	0.077*		–0.045	–0.051	−0.069*
D-A^2^, *β_5_*			–0.053		–0.029	–0.023		0.029	0.038	0.044
Job satisfaction						0.118*			0.180***	0.153**
Work engagement										0.226***
Congruence line	Slope: β_1_+β_2_	–	0.678***	–	0.804***	0.722***	–	0.694***	0.572***	0.408***
	Curvature: β_3_+β_4_+β_5_	–	–0.008	–	0.035	0.036	–	0.045	0.046	0.038
Incongruence line	Slope: β_1_−β_2_	–	0.424***	–	–0.092	−0.142*	–	0.126*	0.050	0.082
	Curvature: β_3_−β_4_+β_5_	–	–0.076	–	−0.127**	−0.118**	–	0.135**	0.148***	0.175***
	R^2^	0.508	0.514	0.617	0.624	0.631	0.548	0.561	0.579	0.602

*,

**, and

*** indicate significance at p < 0.05, p < 0.01, and p < 0.001, respectively.

#### Effects of Need-Supply Fit and Demands-Abilities Fit on Job Satisfaction

**FIGURE 1:**
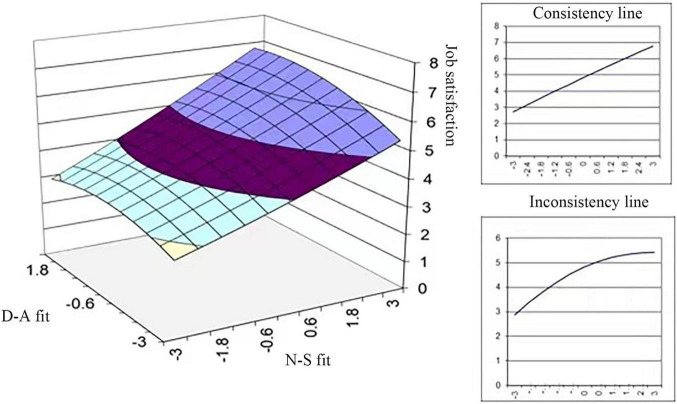
Effects of P-E fit on job satisfaction.

Model 1 and Model 2 describe the linear effects and quadratic effects of N-S fit and D-A fit on job satisfaction, respectively. The results show that N-S fit and D-A fit are significantly and positively related to port employees’ job satisfaction and that the impact of N-S fit is much greater than that of D-A fit (β_1_ = 0.553^***^ > β_2_ = 0.124^***^, *p* < 0.001). This indicates that satisfying port employees’ needs is more important for their job satisfaction. The effects of N-S fit and D-A fit on job satisfaction have a significant slope and an insignificant curvature on the congruence line and incongruence line, which means that the quadratic effect of N-S fit and D-A fit on job satisfaction is insignificant. It can be concluded that the influence of N-S fit and D-A fit on the job satisfaction of employees in port companies does not have maximal value and that port companies should make a long-term commitment to focusing on the N-S fit and D-A fit of port employees. Based on the analysis above, hypothesis H1a and Hypothesis H1b are not supported, as shown in Figure 1.

#### Effects of Need-Supply Fit and Demands-Abilities Fit on Work Engagement

The results of Model 3 indicate that N-S fit and D-A fit are significantly and positively related to the work engagement of port employees and that the impact of N-S fit is smaller than that of D-A fit (β = 0.384^***^ < β = 0.432^***^, *p* < 0.001). This indicates that D-A fit plays a more important role in increasing the work engagement of individuals in port companies. Model 4 shows that the slope is significant and the curvature is insignificant on the congruence line, while the result is the opposite on the incongruence line. This means that when the N-S fit and D-A fit are inconsistent, their influences on work engagement present an inverted U shape. The results reveal that the positive impacts of P-E fit on port employees’ work engagement are likely to decrease after reaching a maximal value due to factors more closely associated with D-A fit. This may be because port employees feel tired after a long day of hard work when they are confronted with high job demands (e.g., workload, emotional demands, and mental demands) (Bakker and Demerouti, 2008), which reduces their work engagement. For example, stevedores have a heavy workload each day due to constant and repeated handling operations, and inspections and management of storage require a high level of dedication from employees because of the responsibilities they entail. Based on the above results, hypothesis H2a and hypothesis H2b are supported.

**FIGURE 2:**
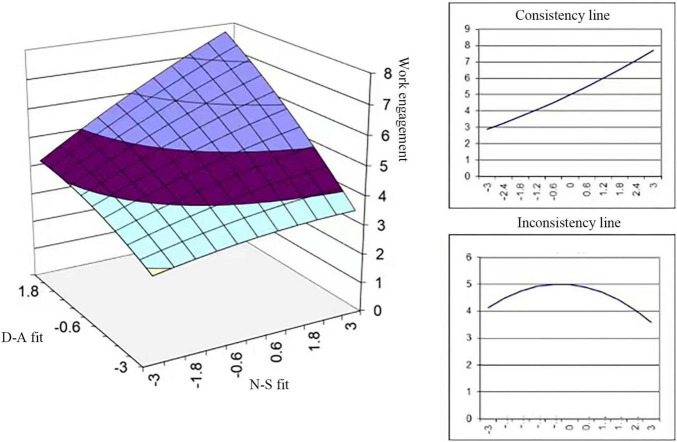
Effects of P-E fit on work engagement.

The results of Model 5 show that the positive quadratic influence of N-S fit and D-A fit on work engagement is significant (β_1_ = 0.290^***^, β_2_ = 0.432^***^, and *p* < 0.001) with job satisfaction’s significant mediating effect (β = 0.118*, *p* < 0.05), which supports hypothesis H4 (as shown in Figure 2). It is worth noting that D-A fit is more important (β_1_ = 0.290^***^ < β_2_ = 0.432^***^, *p* < 0.001). This indicates that providing learning opportunities and building a workforce with futureproof skills to respond to new and changing demands in the context of the digital transformation of ports could improve their job satisfaction and thereby boost employees’ work engagement more effectively.

#### Effects of Need-Supply Fit and Demands-Abilities Fit on Innovation Performance

Model 6 and Model 7 describe the impacts of N-S fit and D-A fit on the innovation performance of port employees. Both N-S fit and D-A fit are significantly and positively related to innovation performance (β_1_ = 0.368^***^, β_2_ = 0.327^***^, and *p* < 0.001). By pioneering new business models and realizing the benefits of new digital and automated processes, ports can maximize the throughput of goods with seamless onward connections. The results reveal that continuing professional training and development allows port employees to update their abilities in line with technological advances, and satisfying the diverse needs of different employees or different needs at different stages of the career development of an individual plays a more important role in promoting the innovation performance of port staff. In Model 7, the slope is significant and the curvature is insignificant on congruence line, and both slope and curvature are significant on the incongruence line, which means the impacts of N-S fit and D-A fit on innovation performance are both U-shaped, with the minimum value appearing when they are inconsistent. This may be because there is a time lag between P-E fit and employees’ innovation performance. From the perspective of D-A fit, the digitization of the departure and arrival of ships, dock planning and cargo handling increases the demand for employees with highly specialized skills such as technology and engineering. However, only a small portion of port employees are highly educated (7.4% had a master’s degree in this study), and this group needs more time to conform with future skill needs. The needs of port employees are highly diversified, which makes it more difficult to coordinate N-S fit than D-A fit. For instance, administrative staff focus on career development; employees in the front-line operation posts care about not only their income and welfare but also work safety; people who are not local expect the improvement of logistics support; and young local employees expect paid holidays and a good work environment. Therefore, the positive impact of P-E fit manifests in the long run despite the decreasing trend during the early stages. Based on the results of Model 6 and Model 7, hypothesis H3a and hypothesis H3b are supported.

**FIGURE 3:**
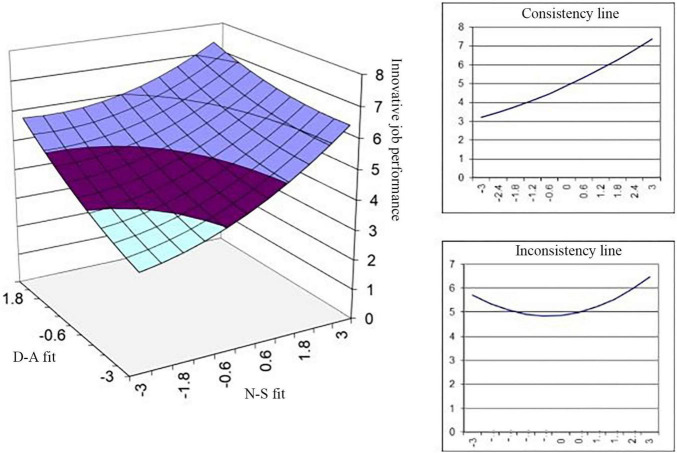
Effects of P-E fit on innovation performance.

Model 8 and Model 9 describe the significant mediating effects of job satisfaction (β = 0.180^***^, *p* < 0.001) and work engagement (β = 0.226^***^, *p* < 0.001), respectively. The results show that the quadratic effects of N-S fit and D-A fit on the innovation performance of port employees are significant with the mediating effect of work engagement (β_1_ = 0.245^***^, β_2_ = 0.163^***^, and *p* < 0.001), which supports hypothesis H5 (as shown in Figure 3). This means that promoting work engagement contributes to the improvement of port employees’ innovation performance in the process of P-E fit management and that N-S fit makes more contributions in this process.

#### The Mediating Effect of Job Satisfaction

Bootstrapping was used to illustrate the mediating role of job satisfaction and work engagement more scientifically (Baron and Kenny, 1986). If the result interval does not contain 0, the mediating effect is significant; otherwise, it is insignificant.

Table 4 shows that the direct and indirect effects of N-S fit and D-A fit on work engagement are significant. The direct and indirect effects of the square term of N-S fit are insignificant, while the direct effect of the square term of D-A fit is significant. The direct and indirect effects of the product term of D-A fit and N-S fit on work engagement are significant. Based on the above results, it can be concluded that job satisfaction has a partial mediating effect on the inverted U-shaped influence of N-S fit and D-A fit on port employees’ work engagement. Hypothesis H4 is therefore confirmed.

**TABLE 4:** Test results of job satisfaction’s mediating effect.

	Work engagement				
	
	Model 1	Model 2	Model 3	Model 4	Model 5
**Direct effect**					

N-S, *β_1_*	0.487 (0.395, 0.578)				
D-A, *β_2_*		0.517 (0.442, 0.592)			
N-S^2^, *β_3_*			0.030 (−0.011, 0.071)		
N-S × D-A, *β_4_*				0.077 (0.022, 0.133)	
D-A^2^, *β_5_*					0.056 (0.004, 0.109)

**Indirect effect**					

Job satisfaction	0.124 (0.056, 0.198)	0.151 (0.106, 0.206)	−0.007 (−0.048, 0.037)	0.056 (0.003, 0.113)	0.007 (−0.038, 0.062)
R-sq	0.506***	0.571***	0.365***	0.374***	0.369***
F	76.439	99.477	42.946	44.618	43.669

*** indicates significance at the level of p < 0.001.

#### The Mediating Effects of Work Engagement

As shown in Table 5, both the direct and indirect effects of N-S fit and D-A fit on the innovation performance of port employees are significant. The quadratic effect parameter of N-S fit has a significant direct effect but an insignificant indirect effect, and the same is true for the square term of D-A fit. The product term of D-A fit and N-S fit has no direct effect on the innovation performance of port employees, while the indirect effect mediated by work engagement is significant. In summary, work engagement has a partial mediating effect on the U-shaped relationship between N-S fit, D-A fit and port employees’ innovation performance. Thus, Hypothesis H5 is confirmed.

**TABLE 5:** Test results of the meditating effect of job satisfaction and work engagement.

	Innovation performance				
	
	Model 6	Model 7	Model 8	Model 9	Model 10

Direct effect					
N, *β_1_*	0.231 (0.143, 0.320)				
D-A, *β_2_*		0.041 (0.010, 0.073)			
N-S^2^, *β_3_*			0.041 (0.010, 0.073)		
N-S × D-A, *β_4_*				0.007 (−0.038, 0.051)	
D-A^2^, *β_5_*					0.049 (0.008, 0.090)

**Indirect effect**					

Total Indirect effect	0.309 (0.226, 0.394)	0.315 (0.244, 0.414)	0.007 (−0.044, 0.067)	0.087 (0.017, 0.157)	0.031 (−0.025, 0.105)
Job satisfaction	0.101 (0.032, 0.169)	0.117 (0.067, 0.178)	−0.004 (−0.027, 0.021)	0.028 (−0.001, 0.061)	0.003 (−0.018, 0.033)
Work engagement	0.208 (0.144, 0.273)	0.198 (0.123, 0.285)	0.010 (−0.022, 0.052)	0.059 (0.014, 0.113)	0.028 (−0.012, 0.081)
R-sq	0.487***	0.564***	0.535***	0.527***	0.533***
F	88.783	80.084	71.227	68.960	70.896

*** indicates significance at the level of p < 0.001.

## Conclusion and Discussion

### Conclusion

This study uses polynomial regression with response surface analysis to explore the quadratic effect of N-S fit and D-A fit on job satisfaction, work engagement and innovation performance among port employees, and the mediating role of job satisfaction and work engagement in the relationship between N-S fit and D-A fit and innovation performance are tested by bootstrapping. The following conclusions are drawn: (1) N-S fit and D-A fit improve port employees’ job satisfaction, work engagement and innovation performance, and the impacts on work engagement and innovation performance are inverted “U” and “U” shaped, respectively; (2) job satisfaction has a mediating effect on the quadratic effect of P-E fit on work engagement, and D-A fit is more important in the process; and (3) work engagement mediates the quadratic effect of P-E fit on the innovation performance of port employees, and N-S fit makes a greater contribution.

In sum, this is the first study on port human resource management to examine the non-linear effects of N-S fit and D-A fit on job satisfaction, work engagement and innovation performance and test the mediating effect of job satisfaction and work engagement on the links between P-E fit and innovation performance.

### Practical Implications

Person-environment fit theory predicts that a correspondence between a person and environmental dimensions will result in positive outcomes such as job performance and job satisfaction. This study emphasizes the importance of P-E fit for managing employees’ job satisfaction, work engagement, and innovation performance in port companies and has several management implications.

Firstly, the results reveal that D-A fit is more important to promoting the work engagement of port employees with the mediating effect of job satisfaction. Therefore, it is crucial to make efforts to educate graduates with appropriate and needed skills that fully conform with future skills needs to respond to the new and changing training needs in the port sector. Digitalization and more advanced communications will lead to better connectivity, greater efficiency and cost savings. Accordingly, port companies should reform their training and education activities according to the requirements of different posts. For example, skill sets of functional competencies are crucial for operational staff. Operations monitoring and analysis, equipment operation and control equipment maintenance and repair are highly important to managing the critical operations of cargo in a safe manner. Additionally, behavioral competencies such as communication skills, teamwork skills, leadership and language ability are important for employees with operation and management roles. Additionally, port staff are expected to be professional and to exhibit ethical behavior, discipline and responsibility in the process of terminal handing, and methodological abilities including creating and innovating, learning and resourcing, and managing complexity are also significant for all employees.

Secondly, since job satisfaction mediates the positive influence of P-E fit on work engagement, work engagement mediates the impact of P-E fit on the innovation performance of port staff, and N-S fit plays a more important role in both processes, innovation performance could be improved by increasing job satisfaction to create a dedicated workforce. The work itself, wages and promotions, working conditions, colleagues and superiors, and the match between work and personality can all affect job satisfaction (Ukil, 2016). Port companies could align ratings training and apprenticeships with industry needs to satisfy the career growth needs of employees. Additionally, incentive compensation systems should be established and broadband salary structures could be explored to promote firm’s learning culture. Moreover, providing a supportive work environment and encouraging communication between colleagues could generate a motivational process leading to employee engagement (Tripathi et al., 2021). Last, mapping career paths and building professional development into training programs allows a proactive approach to career planning according to personal characteristics, and cross-sector mobility should be supported in line with personal growth and progress.

Thirdly, the overall effect of P-E fit should be coordinated. The results indicate that the effects of both N-S fit and D-A fit have a maximal value for work engagement and a reciprocal U curve for the innovation performance of port employees. By contrast, there is a positive linear relationship between P-E fit and job satisfaction. Therefore, with the non-linear incremental effects of N-S fit and D-A fit on work engagement, port companies need to focus on balancing the heavy workload and guaranteeing moderate working hours for employees, since engaged employees are not addicted to their work (Bakker and Demerouti, 2008). In addition, port companies need to understand that the positive effects of N-S fit and D-A fit on employees’ innovation performance increase at a later stage, so they should not give up on P-E fit efforts. Additionally, port companies should be devoted to improving people’s job satisfaction, and more attention should be given to N-S fit due to the positive linear correlation between P-E fit and job satisfaction. Overall, managers in port companies should obtain a thorough understanding of the specific content and comprehensive effects of P-E fit on organizational management and systematically and dynamically make reasonable human resource management plans and improve staff quality based on P-E theory.

### Limitations and Future Research

This study also has certain limitations. The data are cross-sectional, and longitudinal data are recommended to explore long-term trends in the future. Second, the present study relied on self-judgments to measure the innovation performance and work engagement of employees. There is always the potential for bias in perceptual processes. Therefore, future research might address this issue by categorizing supervisors and peers according to their positions or departments to measure work engagement and innovation performance, and grouped regression could be carried out to provide more targeted implications.

## Data Availability Statement

The raw data supporting the conclusions of this article will be made available by the authors, without undue reservation.

## Author Contributions

DJ: design work, including of the model, hypothesis, survey, etc. TL: data analysis. ZC, XZ, and TH: translation and language polish. SW: collating references. LN: organization. All authors contributed to the article and approved the submitted version.

## Conflict of Interest

The authors declare that the research was conducted in the absence of any commercial or financial relationships that could be construed as a potential conflict of interest.

## Publisher’s Note

All claims expressed in this article are solely those of the authors and do not necessarily represent those of their affiliated organizations, or those of the publisher, the editors and the reviewers. Any product that may be evaluated in this article, or claim that may be made by its manufacturer, is not guaranteed or endorsed by the publisher.
